# Sociodemographic and clinical factors associated with receipt of biomarker testing in patients with metastatic colorectal cancer

**DOI:** 10.1002/cam4.4995

**Published:** 2022-07-15

**Authors:** Sarah C. Markt, Benjamin D. Booker, Wyatt Bensken, Nicholas K. Schiltz, Fredrick R. Schumacher, Johnie Rose, Greg Cooper, J. Eva Selfridge, Siran M. Koroukian

**Affiliations:** ^1^ Department of Population and Quantitative Health Sciences Case Western Reserve University School of Medicine Cleveland Ohio USA; ^2^ Case Comprehensive Cancer Center Case Western Reserve University School of Medicine Cleveland Ohio USA; ^3^ Francis Payne Bolton School of Nursing Case Western Reserve University Cleveland Ohio USA; ^4^ Department of Internal Medicine University Hospitals Cleveland Medical Center Cleveland Ohio USA; ^5^ Division of Solid Tumor Oncology University Hospitals Cleveland Medical Center Cleveland Ohio USA

**Keywords:** biomarker, disparities, metastatic colorectal cancer

## Abstract

**Background:**

Standard clinical practice and national guidelines dictate somatic testing of metastatic colorectal cancer (mCRC) tumors to guide appropriate therapy; however, previous studies suggest that not all patients are tested. The objective of this study was to investigate potential differences in testing for mCRC by demographic and clinical factors.

**Methods:**

We performed a retrospective review of de‐identified patient data derived from electronic health records (EHRs) of 25,469 patients diagnosed with mCRC between the years 2013 and 2020. Our outcome was a receipt of the following tests: (a) biomarker testing (BRAF, KRAS, NRAS, MMR/MSI) and (b) next‐generation sequencing (NGS). We interrogated our data using the machine‐learning algorithm Classification and Regression Tree (CART), a unique approach to identifying combinations of, rather than individual demographic and clinical characteristics associated with receipt of testing.

**Results:**

A total of 25,469 patients were identified with mCRC. Of these, 21,133 (83%) received either biomarker testing only (*n* = 12,485) or any testing (biomarker + NGS) (*n* = 8648). The proportion of patients who received any testing increased over calendar time for all age, race, and sex categories. Receipt of any testing was highest (90%) among younger and patients with better performance status, and there was no difference in receipt of any testing by race. The highest percentage of NGS testing was among those with better performance status, <70 years old, commercial or other governmental program payers, and low comorbidity burden; however, those who were Black or Hispanic had a lower prevalence of NGS testing than those who were White.

**Conclusions and Relevance:**

Considerable variations exist in somatic biomarker testing across subgroups of the population. Identification of genomic alterations can aid in determining targeted treatment and improving clinical outcomes; therefore, equitable use of these testing strategies, particularly NGS, is necessary.

## Graphical abstract

The objective of this study was to investigate potential differences in biomarker testing for mCRC by demographic and clinical factors. Identification of genomic alterations can aid in determining targeted treatment and improve clinical outcomes; therefore, equitable use of these testing strategies, particularly NGS, is necessary.

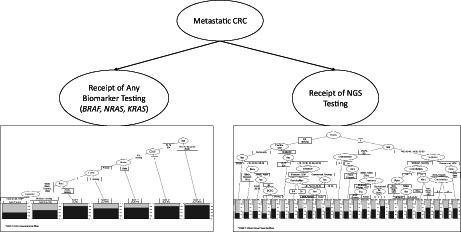

## 1 BACKGROUND

Colorectal cancer (CRC) is the third most common cancer in men and women. There will be an estimated 149,000 new cases of CRC diagnosed in 2021 and over 52,000 deaths.^1^ Despite concerted efforts to improve CRC care across all subgroups of the population, diagnosis, staging, and mortality disproportionally impacts racial and ethnic minorities and people from low socioeconomic backgrounds. In addition, mounting evidence further supports inequities in receipt of potentially curative treatment and survival.^2^, ^3^, ^4^, ^5^, ^6^, ^7^

Precision oncology through targeted therapy has the potential to greatly improve morbidity and mortality. However, the use of pathologic or molecular biomarker testing, broadly referred to as biomarker testing, is a critical first step in diagnostic evaluation to inform the selection of specific treatment. In addition, there is increased interest in the utilization of next‐generation sequencing (NGS) to identify patients to match with clinical trials.^8^, ^9^

The use of such testing, if not distributed equitably, has the potential to further exacerbate existing disparities. Current National Comprehensive Cancer Network (NCCN) guidelines state that all newly diagnosed colon and rectal cancer patients should undergo tumor testing, including microsatellite instability (MSI) or mismatch repair (MMR) testing.^10^ In addition, those with metastatic colon cancer should have tumor somatic genotyping for *KRAS*, *NRAS*, and *BRAF* mutations.^8^, ^10^

Like many other state‐of‐the‐art cancer management strategies, the uptake of biomarker testing likely varies greatly across subgroups of the population, explained in part by racial/ethnic and socioeconomic disparities, including insurance status, as well as differences based on where patients receive their care (e.g., community vs. academic hospital). Using all‐payer, de‐identified data derived from electronic health records (EHRs) generated from routine clinical care across the United States, we aim to (1) examine temporal trends in the uptake of biomarker testing by age, sex, race/ethnicity, and performance status and (2) identify combinations of demographic and clinical characteristics associated with tumor somatic biomarker testing using Classification and Regression Tree (CART) analysis.

CART is a data‐mining technique that allows us to characterize patterns of demographic and clinical characteristics associated with receipt of biomarker testing. This novel machine‐learning analytic approach is a sharp departure from prior studies, which have analyzed one factor at a time. Rather than focusing on individual characteristics, CART identifies subgroups of the population experiencing the outcome, thus allowing us to identify combinations of variables associated with higher and lower rates of testing.^11^, ^12^, ^13^, ^14^, ^15^ The CART approach allows us to characterize phenotypes of biomarker testing with the results visualized as a tree, permitting a more intuitive interpretation of complex interactions between factors.

We hypothesize that although there has been significant uptake over time in biomarker testing across all demographic subgroups of the population, significant disparities persist across groups based on combinations of age, sex, race/ethnicity, comorbidity burden, and Eastern Cooperative Oncology Group (ECOG) performance status.

## 2 METHODS

### 2.1 Study population

We used the nationwide Flatiron Health EHR‐derived database, comprising de‐identified data on cancer patients from approximately 800 sites of care from 280 unique clinics across the United States, with over 90% of practices being community‐based. We identified 25,500 patients who were diagnosed with mCRC during the years 2013–2020, with at least 6 months of follow‐up. We further excluded those with missing de novo metastatic status (*n* = 29) or sex (*n* = 2). Our final analytic study population (*n* = 25,469) included patients diagnosed with localized CRC that later progressed to metastatic disease and those who were de novo metastatic. Our study was approved as not research involving human subjects by the Institutional Review Board at Case Western Reserve University (IRB 2020‐1000).

### 2.2 Outcomes of Interest

We identified documented receipt of the following tests: (a) biomarker testing as defined by receipt of BRAF, KRAS, NRAS, MMR/MSI testing through immunohistochemistry (IHC), Fluorescence in situ hybridization (FISH), or polymerase chain reaction (PCR) and (b) NGS. Some patients received neither, whereas others received both biomarker testing and NGS. Our primary outcome of interest was a receipt of any testing (biomarker and/or NGS) at any time in their record. As a secondary analysis, we evaluated receipt of NGS, compared with biomarker testing, among those with documented tests. We also conducted a sensitivity analysis to compare receipt of any testing 6 months after mCRC diagnosis.

### 2.3 Independent variables

Demographic variables included age at metastatic diagnosis (<40, 40–49, 50–59, 60–69, 70–79, and 80+) and sex (male or female). We combined race and ethnicity (White, Black, Asian, Hispanic, and Other). The payer category (Commercial, Medicare, Other Government Program [OGP], and Unknown) at diagnosis was determined by the payer with a start date before metastatic diagnosis and with an end date after metastatic diagnosis date or no end date listed. The practice setting was described as community or academic. To characterize the health status, we used the ECOG Performance Status (0–4) in 6 months prior to and after metastatic diagnosis, with higher values indicating sicker patients, and categorized as 0/1 (little to no impairment), 2 (some impairment), 3/4 (high impairment), and missing. For those with multiple ECOG scores during this time, we took the median value and rounded up. In addition, using ICD‐9 and ICD‐10 diagnosis codes, a total of 28 comorbid conditions as defined by Elixhauser et al. were identified from encounters occurring 6 months prior to or after diagnosis. Clinical characteristics included the anatomic site of the tumor (colon vs rectum), and whether the patient presented with metastatic disease at diagnosis. Finally, to account for temporal trends, and the expectation that biomarker testing and NGS would become more ubiquitous over time, we included the year of metastatic diagnosis (categorical) in our models.

### 2.4 Statistical analysis

We compared documented receipt of any testing and NGS by clinical and sociodemographic factors using medians and interquartile range values for continuous variables and percentages for categorical variables. We calculated the fraction of patients receiving testing and NGS by year of metastatic diagnosis, overall and by race/ethnicity, sex, age, and ECOG. The denominator included patients with a metastatic diagnosis date in the corresponding year, and the numerator included those with documented testing at any time. The prevalence of testing in 2020 is restricted to those who had documented testing before March 2020 due to the 6‐month follow‐up inclusion criteria.

To identify combinations of demographic and/or clinical factors associated with testing, we used modern generalizations of the CART models.^16^ As a nonparametric, machine‐learning approach, CART uses recursive partitioning of the values of each predictor variable into two sets, such that the values of the outcome variable are as homogeneous as possible in each set. The CART approach begins with the parent node, which includes all the data. Following this, the higher nodes represent the most important variables associated with outcome prediction. Each predictor variable is considered for a potential split. The optimal split is the one that yields the largest reduction in the impurity index, which is a measure of the extent of misclassification at a given node. We used the following stopping criteria to define our trees: a maximum tree depth of six splits and a minimum node size of 25 patients, determining node splits based on the impurity index and requiring each split to increase the complexity parameter by at least 0.00001. Because we were not interested in predictive modeling, we did not include cross‐validation in our primary analyses; however, in sensitivity analyses, we utilized more conservative complexity parameters and conducted cross‐validation, and results were similar (data not shown). To further evaluate factors associated with receipt of any testing, or receipt of NGS within subgroups, we conducted multivariable logistic regression models within subgroups defined by the high or low prevalence of testing.

We used R version 4.0.3 for all statistical analysis, including the ‘rpart’ (CART), ‘partykit’ (tree graphics), and ‘caret’ (for model tuning and cross‐validation) packages. For all models, missing values were modeled using the missing indicator approach, with the *p* value significance threshold set at *p* < 0.05.

## 3 RESULTS

Of the 25,469 patients with mCRC, 21,133 (83%) received either biomarker only (*n* = 12,485) or biomarker + NGS testing (*n* = 8648). Among the 21,133 patients tested, the median time to testing from metastatic diagnosis was 6 weeks (IQR: 2–23 weeks).

### 3.1 Demographic characteristics of receipt of any testing and receipt of NGS

Those who received any testing were younger, more likely to be male, and have a commercial health plan, compared with those who did not receive testing (Table 1). For clinical characteristics, those with lower (0/1) ECOG scores were more likely to receive testing; the percentage of patients with a missing ECOG score was higher among those not tested than those who were tested (47% vs 38%). In addition, those with colon cancer, rather than rectal, and with a lower comorbidity burden, compared with high, were also more likely to be tested.

**TABLE 1:** Demographic and clinical characteristics of patients with mCRC by biomarker testing status

	Not tested, *N* = 4336, *n* (%)	Tested, *N* = 21,133, *n* (%)
Age at metastatic diagnosis		
<40	58 (1.3)	830 (3.9)
40–49	203 (4.7)	2344 (11.1)
50–59	637 (14.7)	4772 (22.6)
60–69	1065 (24.6)	5952 (28.2)
70–79	1574 (36.3)	5352 (25.3)
80+	799 (18.4)	1883 (8.9)
Race		
White	2733 (63.0)	13,601 (64.4)
Black or African American	444 (10.2)	2236 (10.6)
Asian	111 (2.6)	629 (3.0)
Hispanic or Latino	240 (5.5)	1062 (5.0)
Other race	394 (9.1)	1775 (8.4)
Missing race	414 (9.5)	1830 (8.7)
Community practice type	4002 (92.3)	19,530 (92.4)
Female	2047 (47.2)	9446 (44.7)
Primary site		
Colon	3070 (70.8)	15,704 (74.3)
Rectum	1140 (26.3)	4997 (23.6)
Not specified	126 (2.9)	432 (2.0)
Payer type		
Commercial health plan	1272 (29.3)	6777 (32.1)
Medicare	609 (14.0)	2285 (10.8)
Other governmental program	262 (6.0)	1123 (5.3)
Unknown	2193 (50.6)	10,948 (51.8)
Year of metastatic diagnosis		
2013	799 (18.4)	2426 (11.5)
2014	777 (17.9)	2728 (12.9)
2015	732 (16.9)	3040 (14.4)
2016	707 (16.3)	2988 (14.1)
2017	488 (11.3)	3169 (15.0)
2018	424 (9.8)	3153 (14.9)
2019	334 (7.7)	3023 (14.3)
2020	75 (1.7)	606 (2.9)
ECOG category		
0/1 (little to no impairment)	1594 (36.8)	11,189 (52.9)
2 (some impairment)	484 (11.2)	1537 (7.3)
3 (high impairment)	234 (5.4)	408 (1.9)
Missing	2024 (46.7)	7999 (37.9)
De Novo metastatic	2336 (53.9)	12,149 (57.5)
More than five comorbidities	422 (9.7)	2381 (11.3)

Among patients who received testing, those who underwent NGS were more likely to be young, White, commercially insured, and seen at an academic center (Table S1). Conversely, we observed a lower percentage of older patients, Blacks, and Hispanics, among those who received biomarker testing + NGS (Table S1).

### 3.2 Time trends

**FIGURE 1:**
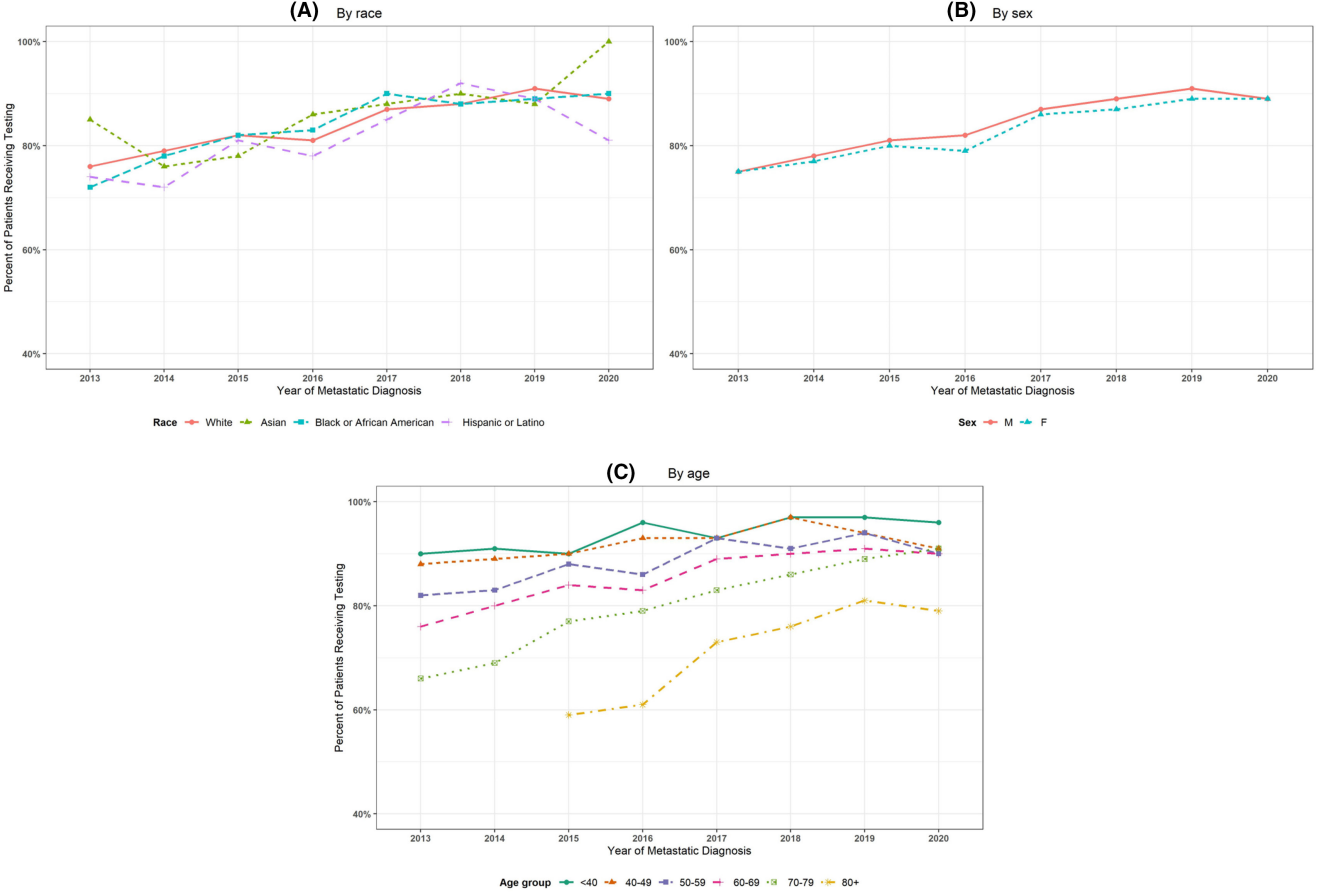
Percent of patients who received any genomic testing over calendar time by (A) race, (B) sex, and (C) age at metastatic diagnosis.

**FIGURE 2:**
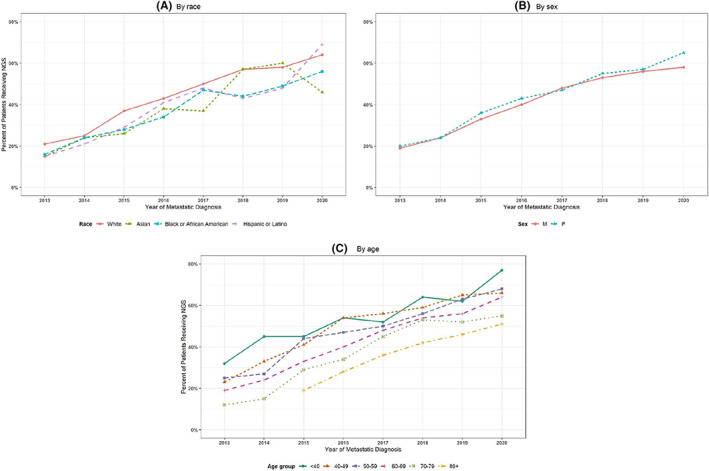
Percent of patients who received next‐generation sequencing over calendar time by (A) race, (B) sex, and (C) age at metastatic diagnosis.

The proportion of patients who received any testing increased over the calendar time of metastatic diagnosis for all age, race, and sex categories (Figure 1A–C). In addition, the proportion remained similar by race categories over time. Similarly, among patients who received any testing, the proportion of those sent for NGS increased from 19% in 2013 to 56% in 2019. There were no differences in the proportion tested over time by sex (unadjusted chi‐square *p* = 0.18). Black patients were less likely to receive NGS regardless of the year of diagnosis (unadjusted chi‐square *p* < 0.01), with an 8% difference in testing between white and black patients in 2019 (Figure 2A–C). We similarly evaluated trends in any testing and NGS by ECOG score (Figure S1). Furthermore, we found that adjusting for other factors, regardless of year, older, higher ECOG, and racially diverse patients were less likely to receive NGS, compared with their counterparts (Table S2).

### 3.3 Receipt of any testing

**FIGURE 3:**
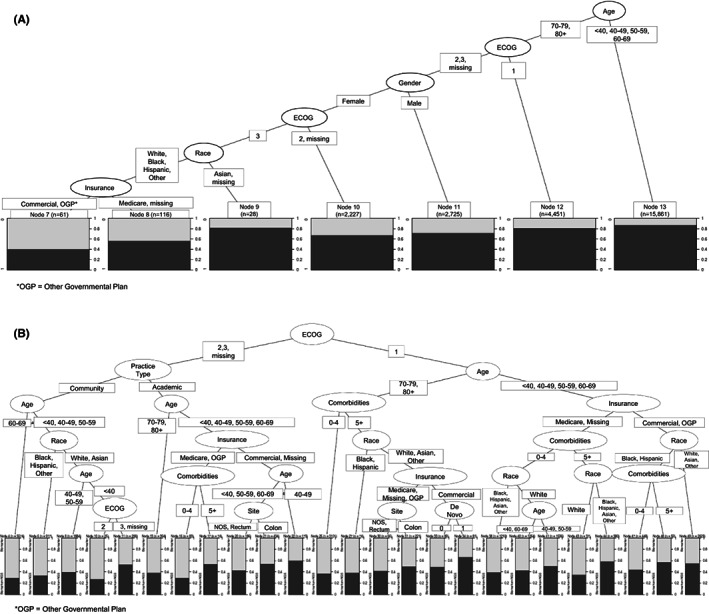
CART model for receipt of: (A) any testing and (B) next‐generation sequencing.

The CART analysis to examine factors associated with receipt of testing showed that the most important variable was age, and that the group of patients in whom we observed the highest level of testing was that consisting of patients younger than 70 years old (Figure 3). In that group of 15,861 patients, about 85% received testing (node 13). Conversely, the lowest percentage of testing (40%) was observed among patients 70 years of age or older, had ECOG score of 3, female, White, Black, Hispanic or Other race (vs Asian), and had commercial or other governmental insurance (node 7, *n* = 61). Results were similar when we included only patients tested within the 6 months of metastatic diagnosis (data not shown).

Among those patients younger than 70 (node 13), those with rectal (vs colon) tumor site, Hispanic and Other race (vs White), seen in a community center (vs academic), ECOG scores 2 or 3+ or missing (vs ECOG 0/1), and Medicare and OGP payers (vs. commercial health plan) were significantly associated with lower odds of receiving testing (Table 2). Conversely, younger patients were more likely to receive testing. In node 7, where we observed a lower prevalence of testing, we found no association between receipt of testing and race, site, practice type, age, or payer category. Our findings were robust when we included year and site in models as random effects (data not shown).

**TABLE 2:** Association between demographic and clinical characteristics and receipt of any testing, among patients younger than 70 (node 13 in Figure 3)

	Odds ratio (OR)	95% CI		*p* value
Race				
White	Reference	Reference		
Black	0.95	0.81	1.11	0.50
Asian	1.03	0.78	1.40	0.84
Hispanic	0.77	0.63	0.94	0.01
Other	0.82	0.70	0.98	0.02
Missing	0.84	0.71	0.99	0.04
ECOG				
0/1	Reference	Reference		
2	0.50	0.42	0.61	<0.01
3+	0.24	0.18	0.32	<0.01
Missing	0.57	0.51	0.63	<0.01
Primary site				
Colon	Reference	Reference		
Rectum	0.71	0.63	0.78	<0.01
NOS	0.66	0.49	0.89	0.01
Age				
60–69	Reference	Reference		
50–59	1.32	1.19	1.47	<0.01
40–49	2.01	1.71	2.36	<0.01
<40	2.60	1.98	3.46	<0.01
Payer category				
Commercial health plan	Reference	Reference		
Medicare	0.78	0.65	0.94	0.01
OGP	0.65	0.53	0.81	<0.01
Unknown	0.84	0.75	0.94	<0.01
Practice type				
Academic	Reference	Reference		
Community	0.83	0.69	0.99	0.04

### 3.4 Receipt of NGS

Among those with any testing, the patients with the highest percentage of NGS testing were among those with low ECOG scores, ages younger than 70 years, and commercial or OGP payers (node 45, *n* = 3130) (Figure 3B). Among these patients, however, those who were Black or Hispanic race with low comorbidity burden (0–4) had a lower prevalence of NGS testing than those who were White, Asian, or Other (42% vs 59%). Black (OR: 0.68, 95% CI: 0.0.54–0.85) and Hispanic race patients (OR: 0.48, 95% CI: 0.34–0.68), compared with White patients, were statistically significantly less likely to receive NGS.

The lowest percentage of NGS testing (30%) was observed among patients with high or missing ECOG scores, receiving care in community practice settings who were older than 60 years of age (node 4). Restricting to patients in node 4 (*n* = 5574; 30% tested with NGS), Black patients (OR: 0.73, 95% CI: 0.59–0.90), compared with White, and patients aged 70–79 (OR: 0.74, 95% CI: 0.65–0.84), compared with those 60–69, had lower odds of receiving NGS; age 80+ was associated with a nonstatistically significant lower odds of NGS, compared with 60–69 (Table 3). Among those patients diagnosed in more recent years (2017–2019), we found that regardless of race, older patients and those with higher ECOG scores had the lowest prevalence of receipt of NGS (Table S3). Among those of similar age and ECOG scores, Black patients had a lower prevalence of NGS compared with White patients (Table S3). Importantly, the prevalence of NGS testing among White patients over 80 with ECOG score <2 (46.2%) was similar to that of Black patients younger than 60 with ECOG score <2 (48.4%).

**TABLE 3:** Association between demographic and clinical characteristics and receipt of NGS, among patients with high or missing ECOG scores, receiving care in community practice settings, and older than 60 years (node 4 in Figure 3B)

	Odds ratio (OR)	95% CI		*p* value
Race				
White	Reference	Reference		
Black	0.73	0.59	0.90	<0.01
Asian	0.85	0.57	1.24	0.41
Hispanic	1.24	0.94	1.62	0.13
Other	1.05	0.86	1.27	0.64
Missing	1.12	0.92	1.35	0.27
Sex				
Male	Reference	Reference		
Female	0.97	0.87	1.09	0.65
Metastatic at diagnosis	1.02	0.91	1.15	0.69
Primary site				
Colon	Reference	Reference		
Rectum	1.09	0.94	1.26	0.25
NOS	1.16	0.80	1.65	0.44
Age				
60–69	Reference	Reference		
70–79	0.74	0.65	0.85	<0.01
80+	0.86	0.72	1.03	0.10
Payer category				
Commercial health plan	Reference	Reference		
Medicare	0.83	0.69	1.00	0.05
OGP	0.84	0.63	1.10	0.21
Unknown	0.79	0.69	0.90	<0.01

## 4 DISCUSSION

Using clinical data from across the United States, we sought to identify subgroups of patients with mCRC who are more or less likely to receive biomarker testing or NGS. Overall, the prevalence of biomarker testing from 2013 to 2020 remained stable, whereas NGS testing increased significantly over time. We found that while the use of testing increased over calendar time for all groups, particularly for NGS, older age and compromised performance status (higher ECOG) were associated with a lower likelihood of receiving biomarker testing or NGS.

The findings by race/ethnicity were more nuanced. We found no differences in the prevalence of testing overall across racial/ethnic groups. However, NGS testing was lower in Black patients than in White patients, and while NGS testing increased over time in all racial/ethnic groups, the gap by race/ethnicity persisted in recent years. Previous studies have shown that the identification of tumor alterations can provide targeted therapy, thus improving clinical outcomes.^17^ Indeed, the ability of biomarkers to improve treatment and reduce costs has been previously demonstrated,^18^, ^19^ including sparing patients from futile, potentially toxic and expensive treatment.^13^, ^14^, ^18^ For example, testing mCRC patients for RAS status and treating only patients without RAS mutations with EGFR inhibitors are more cost‐effective than treating all patients without testing.^19^

Despite the increased interest in the utilization of NGS and its utility to identify patients to match with clinical trials,^8^, ^9^ underrepresentation of racially diverse patients in clinical trials has persisted.^20^, ^21^, ^22^, ^23^, ^24^ Our findings, showing disparities in biomarker testing, point to potential further exacerbation of disparities in targeted therapy and clinical outcomes among those with mCRC and in clinical trial participation. Racial differences in receipt of biomarker testing have been shown in earlier studies among early stage breast cancer patients,^15^, ^25^ metastatic lung cancer,^11^, ^26^ and metastatic CRC,^12^ with racially diverse patients with low incomes and/or on Medicaid being less likely to undergo such testing, despite Medicare and Medicaid programs' coverage of biomarker testing.^15^, ^25^, ^27^, ^28^ In addition, similar to our findings, racial disparities in NGS in breast cancer patients have been persistent, despite increasing trends in testing over time.^15^

Biomarker testing, a critical step in diagnostic evaluation to inform the selection of targeted therapy, is now considered the standard practice as part of the diagnostic evaluation in mCRC patients. However, numerous challenges in the implementation of these standards persist.^29^ A study in 814 patients with advanced non‐small‐cell lung cancer treated by 89 community‐based oncologists at 15 sites in New Jersey and Maryland found that 41% of patients did not undergo the recommended biomarker testing.^30^ Challenges listed included coordination of sample handling, long turnaround times, reimbursement for the tests, access to targeted therapy, insufficient tissue, and patient harm resulting from repeat biopsies when the tissue sample was insufficient. More recent studies to identify contemporaneous barriers to biomarker testing are warranted.

To our knowledge, this is the first study to examine biomarker and NGS testing separately, allowing for a more nuanced examination of the factors and combination of factors associated with receiving one or both types of testing. In addition, our study captures data on each biomarker and NGS testing through 2020. The most recent study that is comparable to ours covers data through 2017,^12^ however, significant increases in the prevalence, particularly of NGS testing, have occurred since 2017, highlighting the importance of our findings. Another important strength of this study lies in our use of CART analysis, a unique approach to identify empirically emerging combinations of factors—rather than individual factors—associated with receipt of biomarker testing as well as the most important splitting variables, including patient age, ECOG score, and race. These findings allowed us to subsequently focus on testing rates in specific strata of the population and to determine that among patients with low ECOG scores, NGS testing was as common among White patients over age 70 years (nodes 30–34 in Figure 3A) as it was in Black patients younger than 60 years of age (node 38). From an equity lens, this finding could be concerning if NGS testing enhances the delivery of oncology care to patients, including through access to clinical trials and targeted therapy. On the other hand, we found that older patients and those with higher ECOG scores (i.e., compromised performance status) had lower NGS testing rates than their younger counterparts; and while NGS testing increased over time in older patients and those with high ECOG scores, the prevalence of NGS testing among older patients and those with higher ECOG score remained less than 50% throughout the study period.

Our findings should be interpreted in light of the following limitations: First, our data included sizable proportions of patients with missing values in race/ethnicity (9%), ECOG score (39%), and insurance status (53%), precluding us from conducting more detailed analysis by these variables. We also lacked data on clinical features such as histology and other demographic characteristics such as income and educational attainment, thus limiting our ability to gain better insight into disparities in testing. We also lacked data on tumor sidedness; prior literature has shown that RAS, KRAS, and BRAF mutations occur more frequently among right‐sided colon tumors than left‐sided colon tumors.^31^, ^32^ Second, although we were able to identify comorbid conditions and characterize comorbidity burden based on the count of these conditions, we may not have been able to fully capture the clinical context and extent to which comorbidity burden and ECOG scores affected the use of these tests, especially given the high rate of missingness for ECOG scores. However, in a previous study,^33^ little was gained in the risk adjustment model by adding the ECOG score to the model that already included the Charlson comorbidity score, which is similar to the Elixhauser comorbidity score used in our study. Additionally, the results should be interpreted with caution and may be subject to differential misclassification, as the completeness, accuracy, and count of relevant conditions may vary greatly across care sites with different coding practices. Last, a limitation of the CART approach is the lack of stability in the combinations identified, and therefore, our trees might look different than those generated by other researchers or other populations. However, we conducted sensitivity analyses using different complexity parameters which yielded similar trees.

In conclusion, there has been great progress in biomarker testing across demographic subgroups of patients with mCRC over the last several years. Future studies should be conducted utilizing real‐world data sets to ensure greater utilization across each of these technologies for equitable access and delivery of cancer care. More effort is needed to improve the uptake of NGS, especially among racially diverse patients, to improve the use of targeted therapy, representation in clinical trials, and to ensure equity in achieving the potential of precision oncology.

## AUTHOR CONTRIBUTIONS

SCM, FRS, JR, GC, and SMK: Conceptualization; SCM, BDB, WB, NKS, FRS, GC, and SMK: Methodology; SCM, BDB, GC, and SMK: Data curation; SCM, BDB, WB, NKS, FRS, JR, GC, and SMK: Writing—review and editing; SCM and SMK: Funding acquisition; SCM and SMK: Supervision.

## FUNDING INFORMATION

This work was supported by the American Cancer Society (WIA‐20‐111‐01‐RWIA).

## CONFLICT OF INTEREST

The authors report no relevant disclosures.

## Supporting information


Figure S1

Table S1

## Data Availability

The data for this study were provided to the authors by Flatiron Health, Inc. and are not publicly available. These deidentified data may be made available upon request, and are subject to a license agreement with Flatiron Health; interested researchers should contact DataAccess@flatiron.com to determine licensing terms.

## References

[1] Siegel RL , Miller KD , Fuchs HE , Jemal A . Cancer statistics, 2021. CA Cancer J Clin. 2021;71(1):7‐33. 33433946 10.3322/caac.21654

[2] Robbins AS , Siegel RL , Jemal A . Racial disparities in stage‐specific colorectal cancer mortality rates from 1985 to 2008. J Clin Oncol. 2012;30(4):401‐405. 22184373 10.1200/JCO.2011.37.5527

[3] Polite BN , Dignam JJ , Olopade OI . Colorectal cancer model of health disparities: understanding mortality differences in minority populations. J Clin Oncol. 2006;24(14):2179‐2187. 16682737 10.1200/JCO.2005.05.4775

[4] Baldwin LM , Dobie SA , Billingsley K , et al. Explaining black‐white differences in receipt of recommended colon cancer treatment. J Natl Cancer Inst. 2005;97(16):1211‐1220. 16106026 10.1093/jnci/dji241 PMC3138542

[5] Laiyemo AO , Doubeni C , Pinsky PF , et al. Race and colorectal cancer disparities: health‐care utilization vs different cancer susceptibilities. J Natl Cancer Inst. 2010;102(8):538‐546. 20357245 10.1093/jnci/djq068 PMC2857802

[6] Esnaola NF , Gebregziabher M , Finney C , Ford ME . Underuse of surgical resection in black patients with nonmetastatic colorectal cancer: location, location, location. Ann Surg. 2009;250(4):549‐557. 19730243 10.1097/SLA.0b013e3181b732a5

[7] Jessup JM , Stewart A , Greene FL , Minsky BD . Adjuvant chemotherapy for stage III colon cancer: implications of race/ethnicity, age, and differentiation. JAMA. 2005;294(21):2703‐2711. 16333005 10.1001/jama.294.21.2703

[8] Biller LH , Schrag D . Diagnosis and treatment of metastatic colorectal cancer: a review. JAMA. 2021;325(7):669‐685. 33591350 10.1001/jama.2021.0106

[9] Flaherty KT , Gray RJ , Chen AP , et al. Molecular landscape and actionable alterations in a Genomically guided cancer clinical trial: National Cancer Institute molecular analysis for therapy choice (NCI‐MATCH). J Clin Oncol. 2020;38(33):3883‐3894. 33048619 10.1200/JCO.19.03010 PMC7676882

[10] Messersmith WA . NCCN guidelines updates: management of metastatic colorectal cancer. J Natl Compr Canc Netw. 2019;17(5.5):599‐601. 31117039 10.6004/jnccn.2019.5014

[11] Kehl KL , Lathan CS , Johnson BE , Schrag D . Race, poverty, and initial implementation of precision medicine for lung cancer. J Natl Cancer Inst. 2019;111(4):431‐434. 30576459 10.1093/jnci/djy202 PMC6449167

[12] Sangare L , Delli‐Zotti K , Florea A , Rehn M , Benson AB , Lowe KA . An evaluation of RAS testing among metastatic colorectal cancer patients in the USA. Future Oncol. 2021;17(13):1653‐1663. 33629919 10.2217/fon-2020-1075

[13] Frank M , Mittendorf T . Influence of pharmacogenomic profiling prior to pharmaceutical treatment in metastatic colorectal cancer on cost effectiveness: a systematic review. Pharmacoeconomics. 2013;31(3):215‐228. 23338963 10.1007/s40273-012-0017-2

[14] Blank PR , Moch H , Szucs TD , Schwenkglenks M . KRAS and BRAF mutation analysis in metastatic colorectal cancer: a cost‐effectiveness analysis from a swiss perspective. Clin Cancer Res. 2011;17(19):6338‐6346. 21807639 10.1158/1078-0432.CCR-10-2267

[15] Ko NY , Qureshi MM , Oladeru OT , et al. Racial differences in genomic testing and receipt of endocrine therapy in early‐stage breast cancer. Breast Cancer Res Treat. 2020;184(3):849‐859. 32888137 10.1007/s10549-020-05888-9

[16] Lemon SC , Roy J , Clark MA , Friedmann PD , Rakowski W . Classification and regression tree analysis in public health: methodological review and comparison with logistic regression. Ann Behav Med. 2003;26(3):172‐181. 14644693 10.1207/S15324796ABM2603_02

[17] Cobain EF , Wu YM , Vats P , et al. Assessment of clinical benefit of integrative genomic profiling in advanced solid tumors. JAMA Oncol. 2021;7(4):525‐533. 33630025 10.1001/jamaoncol.2020.7987 PMC7907987

[18] Poste G . Bring on the biomarkers. Nature. 2011;469(7329):156‐157. 21228852 10.1038/469156a

[19] Unim B , Pitini E , De Vito C , D'Andrea E , Marzuillo C , Villari P . Cost‐effectiveness of RAS genetic testing strategies in patients with metastatic colorectal cancer: a systematic review. Value Health. 2020;23(1):114‐126. 31952666 10.1016/j.jval.2019.07.009

[20] Loree JM , Anand S , Dasari A , et al. Disparity of race reporting and representation in clinical trials leading to cancer drug approvals from 2008 to 2018. JAMA Oncol. 2019;5(10):e191870. 31415071 10.1001/jamaoncol.2019.1870 PMC6696743

[21] Murthy VH , Krumholz HM , Gross CP . Participation in cancer clinical trials: race‐, sex‐, and age‐based disparities. JAMA. 2004;291(22):2720‐2726. 15187053 10.1001/jama.291.22.2720

[22] Unger JM , Hershman DL , Osarogiagbon RU , Gothwal A , Anand S , Dasari A , Overman M , Loree JM , Raghav K Representativeness of black patients in cancer clinical trials sponsored by the National Cancer Institute compared with pharmaceutical companies. JNCI Cancer Spectr 2020;4(4):pkaa034. 32704619 10.1093/jncics/pkaa034 PMC7368466

[23] Tharakan S , Zhong X , Galsky MD . The impact of the globalization of cancer clinical trials on the enrollment of black patients. Cancer. 2021;127:2294‐2301. 33682111 10.1002/cncr.33463

[24] Nazha B , Mishra M , Pentz R , Owonikoko TK . Enrollment of racial minorities in clinical trials: old problem assumes new urgency in the age of immunotherapy. Am Soc Clin Oncol Educ Book. 2019;39:3‐10. 31099618 10.1200/EDBK_100021

[25] Cress RD , Chen YS , Morris CR , Chew H , Kizer KW . Underutilization of gene expression profiling for early‐stage breast cancer in California. Cancer Causes Control. 2016;27(6):721‐727. 27097910 10.1007/s10552-016-0743-4 PMC4871729

[26] Wong W , Wu N , Gupta R , Mansfield AS . Utilization trends and factors associated with ROS1 testing among patients with advanced non‐small‐cell lung cancer in US Community practices. Clin Lung Cancer. 2020;22:e470‐e480. 32763067 10.1016/j.cllc.2020.06.019

[27] Guth AA , Fineberg S , Fei K , Franco R , Bickell NA . Utilization of oncotype DX in an Inner City population: race or place? Int J Breast Cancer 2013;2013:653805, 1, 3. 24455283 10.1155/2013/653805 PMC3878389

[28] Orucevic A , Heidel RE , Bell JL . Utilization and impact of 21‐gene recurrence score assay for breast cancer in clinical practice across the United States: lessons learned from the 2010 to 2012 National Cancer Data Base analysis. Breast Cancer Res Treat. 2016;157(3):427‐435. 27206678 10.1007/s10549-016-3833-9 PMC4903105

[29] Levy BP , Chioda MD , Herndon D , et al. Molecular testing for treatment of metastatic non‐small cell lung cancer: how to implement evidence‐based recommendations. Oncologist. 2015;20(10):1175‐1181. 26330460 10.1634/theoncologist.2015-0114 PMC4591939

[30] Gutierrez ME , Choi K , Lanman RB , et al. Genomic profiling of advanced non‐small cell lung cancer in community settings: gaps and opportunities. Clin Lung Cancer. 2017;18(6):651‐659. 28479369 10.1016/j.cllc.2017.04.004

[31] Digiacomo N , Bolzacchini E , Veronesi G , et al. Neuroendocrine differentiation, microsatellite instability, and tumor‐infiltrating lymphocytes in advanced colorectal cancer with BRAF mutation. Clin Colorectal Cancer. 2019;18(2):e251‐e260. 30638691 10.1016/j.clcc.2018.12.003

[32] Bylsma LC , Gillezeau C , Garawin TA , et al. Prevalence of RAS and BRAF mutations in metastatic colorectal cancer patients by tumor sidedness: a systematic review and meta‐analysis. Cancer Med. 2020;9(3):1044‐1057. 31856410 10.1002/cam4.2747 PMC6997095

[33] Dobbins TA , Badgery‐Parker T , Currow DC , Young JM . Assessing measures of comorbidity and functional status for risk adjustment to compare hospital performance for colorectal cancer surgery: a retrospective data‐linkage study. BMC Med Inform Decis Mak. 2015;15:55. 26174550 10.1186/s12911-015-0175-1 PMC4502567

