# Supplementary material for: Sociodemographic and clinical factors associated with receipt of biomarker testing in patients with metastatic colorectal cancer
Source: Cancer Med. 2022 Jul 15;12(2):1850–9. doi: 10.1002/cam4.4995 (PMC9883565; doi:10.1002/cam4.4995)

Supplemental Figure 1. Percent of patients with receipt of a) any testing and b) next-generation sequencing (NGS) over calendar time by ECOG score

a) Any testing

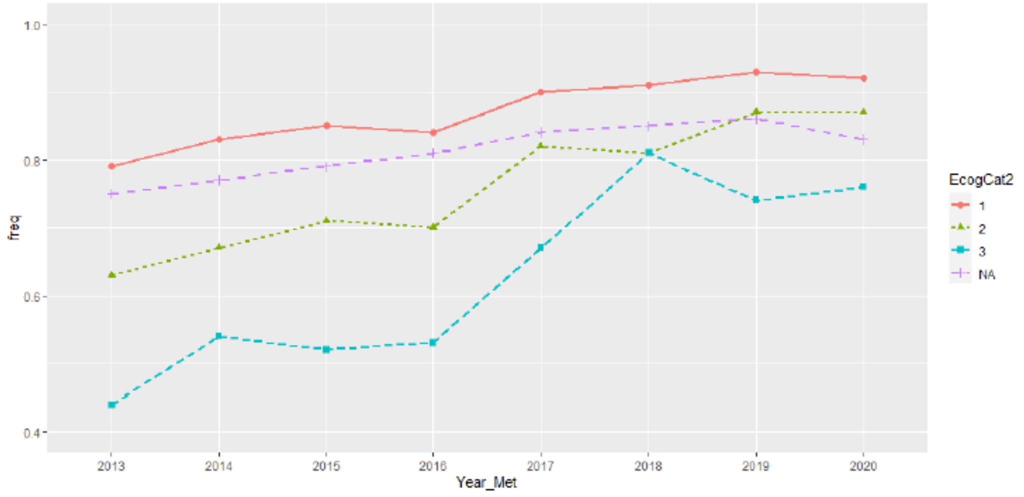

b) NGS

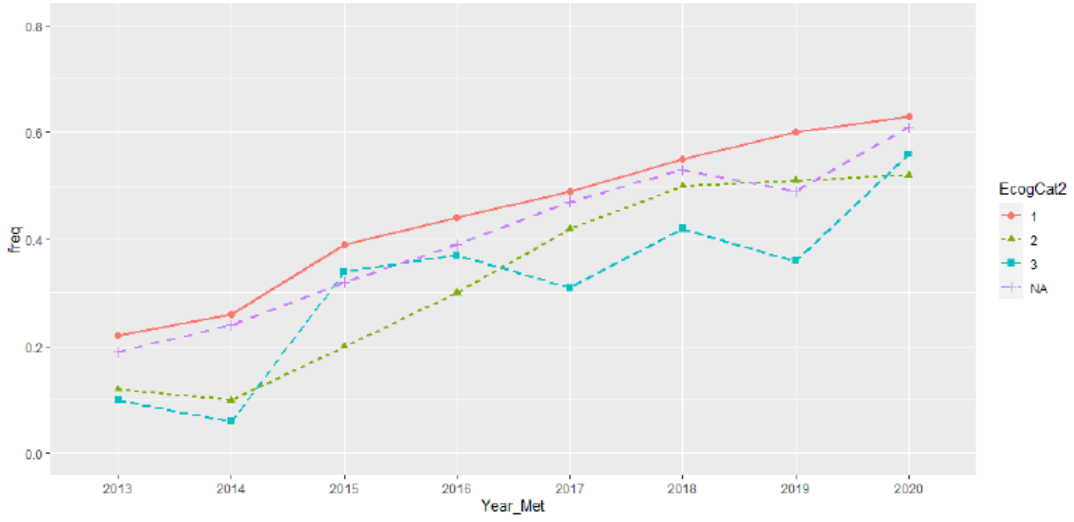

Supplement: Supplementary file 1 — Figure S1 [file CAM4-12-1850-s001.pdf]
